# *“Experiences with disclosure of HIV-positive status to the infected child”*: Perspectives of healthcare providers in Dar es Salaam, Tanzania

**DOI:** 10.1186/s12889-016-3749-7

**Published:** 2016-10-13

**Authors:** Adellah Sariah, Joan Rugemalila, Magreat Somba, Anna Minja, Margareth Makuchilo, Edith Tarimo, David Urassa, Helen Siril

**Affiliations:** 1 Department of Maternal and Child Health, Faculty of Nursing, Hubert Kairuki Memorial University (HKMU), 322 Regent Estate, P.O. Box 65300, Dar es Salaam, +255 Tanzania; 2 Department of Internal Medicine, Muhimbili National Hospital, P.O. Box 65300, Dar es Salaam, Tanzania; 3 Department of qualitative research, African Academy for Public Health (AAPH), P.O. Box 79810, Dar es Salaam, Tanzania; 4 Department of Care and Treatment, Mbagala Rangi Tatu Hospital, P.O. Box 45232, Dar es Salaam, Tanzania; 5 Department of Nursing Management, School of Nursing, Muhimbili University of Health and Allied Sciences (MUHAS), P.O. Box 65004, Dar es Salaam, Tanzania; 6 Department of Community Health, School of Public Health and Social Sciences, P.O. Box 65015, Dar es Salaam, Tanzania; 7 Department of Public Health Evaluation, Management and Development for Health (MDH), P.O. Box 79810, Dar es Salaam, Tanzania

**Keywords:** Disclosure, Paediatric, HIV positive status, Healthcare providers, Parents, Guardians, Tanzania

## Abstract

**Background:**

The specific age to which an HIV infected child can be disclosed to is stipulated to begin between ages 4 and 6 years. It has also been documented that before disclosure of HIV positive status to the infected child. Health care providers should consider children’s cognitive-developmental ability. However, observation and situation analysis show that, health care providers still feel uncomfortable disclosing the HIV positive status to the infected child. The aim of the study was to explore healthcare providers’ experiences in disclosure of HIV-positive status to the infected child.

**Methods:**

A qualitative study involving 20 health care providers who attend HIV-positive children was conducted in September, 2014 in Dar es Salaam, Tanzania. Participants were selected from ten HIV care and treatment clinics (CTC) by purposive sampling. An interview guide, translated into participants’ national language (Kiswahili) was used during in-depth interviews. Sampling followed the principle of data saturation. The interviews focused on perspectives of health-care providers regarding their experience with paediatric HIV disclosure. Data from in-depth interviews were transcribed into text; data analysis followed qualitative content analysis.

**Results:**

The results show how complex the process of disclosure to children living with HIV can be to healthcare providers. Confusion was noted among healthcare providers about their role and responsibility in the process of disclosing to the HIV infected child. This was reported to be largely due to unclear guidelines and lack of standardized training in paediatric HIV disclosure. Furthermore, healthcare providers were concerned about parental hesitancy to disclose early to the child due to lack of disclosure skills and fear of stigma. In order to improve the disclosure process in HIV infected children, healthcare providers recommended further standardized training on paediatric HIV disclosure with more emphasis on practical skills and inclusion of disclosure content that is age appropriate for children with HIV.

**Discussion:**

The disclosure process was found to be a complex process. Perspectives regarding disclosure in children infected with HIV varied among healthcare providers in terms of their role in the process, clear national guidelines and appropriate standardized training for paediatric disclosure. Consistent with other studies, healthcare providers reported difficulties during disclosure because parents /guardians largely fear blame, social stigma, child's negative emotional reaction when disclosed to and have concerns about the child being too young and immature to understand the HIV condition.

**Conclusions:**

In order to prevent inconsistencies during the disclosure process, it is important to have in place clear guidelines and standardized paediatric HIV disclosure training for healthcare providers. This would help improve their skills in paediatric disclosure, leading to positive health outcomes for children infected with HIV.

## Background

Disclosure has been defined as a state in which a child has knowledge about his/her HIV status [1]. With an increase in the use of antiretroviral therapy in low-resource settings, children with HIV/AIDS tend to live longer. Children who are aware of their HIV-positive status have better abilities to seek social support, improved coping skills; [2] and practice safer sexual practices to prevent secondary transmission [3].

Talking to a child about his/her HIV positive status is a challenging task for parents/guardians and healthcare providers. Disclosure has been found to have both psychosocial and health benefits to a child [4]. Healthcare providers should emphasize the importance of parents/guardians in playing their role to disclose to the child. Such disclosure tends to increase a child’s understanding about his/her condition and facilitate active participation in care and treatment. In addition, children are empowered to make the right decisions and learn to protect themselves and others from the disease [5]. However, despite existing guidelines and materials, this process does not happen as required [4]. Evidence from developing countries shows that caregivers lack knowledge, skills and guidance on how to approach HIV-diagnosis disclosure to children [6]. HIV-positive children have indicated that the reasons why their parents or guardians do not talk to them earlier about their HIV status include not knowing enough about the disease symptoms, diagnosis and treatment, fear of stigma and fear that children might not accept the information [5]. It has been found that lower prevalence rates of disclosure are related to fear of stigma and discrimination by the family members who are not aware or/and caregivers who perceive they are not emotionally prepared. They fear that once the HIV-positive status has been disclosed to a child they will tell others leading to stigma and discrimination against the family [7]. Other fears from parents include: fear of isolation, child’s negative emotional reaction such as suicide, guilt and blame related to the perception that HIV is transmitted by promiscuity [5]. Disclosure counseling to parents/guardians is very important because it prepares and enables them to support disclosure in their children [8].

Pediatric HIV infection continues to be a major problem globally. About 3.3 million children below 15 years of age are living with HIV and 2.9 million of these are found in Sub-Saharan Africa [9]. It is estimated that 1,411,829 million Tanzanians are living with HIV; 28 % of these consist of children 0–14 years of age [10]. According to UNAIDS report, only 26 % of children who are eligible receive antiretroviral therapy (ART) in Tanzania [11]. With an increase in the number of children who have access to ART and improved prognosis; disclosure of HIV positive status to an infected child is becoming an increasingly common issue in clinical practice. As these children continue to grow up another important challenge is determining when and how to inform them about their HIV-positive status [12]. Nonetheless, some parents/guardians and healthcare providers are reluctant to inform children about their HIV-positive status [13].

The WHO guidelines on HIV disclosure for children recommends that school age children should be told of their HIV-positive status. Younger children should be informed incrementally to accommodate their cognitive skills and emotional maturity, in preparation for full disclosure. Disclosure should be viewed as a step in the process of adjusting to HIV illness and the life challenges it poses [14]. According to WHO, important elements for inclusion in the paediatric HIV disclosure training for healthcare providers include the following: a choice of culturally appropriate and available resources to assist the disclosure process, communication appropriately geared to children of different ages, providing HIV and its treatment information to parents/guardians and children, preparing parents/guardians for short and long-term emotional reactions of their children,; developing a plan for the child and parent/guardian to disclose to others, preparing parents/guardians to answer questions that children may have over time and to choose “safe” staff members or others with whom to discuss issues, preparing parents/guardians to engage in life-planning with children and reduction of stigma and protection of privacy [14].

The Tanzania National Guidelines for paediatric disclosure recommends that the process of disclosure should begin as early as 4–6 years of age; it should be done overtime. The child can be told that he/she has a chronic disease that needs consistent clinic visits and medications every day. Full disclosure of HIV and AIDS is recommended at about 8–10 years of age in a caring and supporting manner and environment [8]. Despite the existence of guidelines on paediatric HIV disclosure in Tanzania, most healthcare providers still face difficulties during the disclosure process. Observations done by clinical monitors in HIV care clinics in Dar es Salaam reveal that healthcare providers still feel uncomfortable engaging in the paediatric HIV disclosure process even after attending paediatric disclosure training. Furthermore, the current HIV disclosure training curriculum only includes a one hour session with no time for practical and case discussions. This might be limiting acquisition of sufficient skills among healthcare providers to fully support the disclosure of a child’s HIV positive status. Various paediatric HIV trainings have been provided to healthcare providers attending HIV infected children. Most of these trainings are not standardized; they differ in content and duration and the majority have been offered by different health organizations. At the clinic level there is a poor system that supports healthcare provider practices which follow the national guidelines. It is crucial to address the existing gap between paediatric HIV disclosure training and the actual practice among healthcare providers in order to maximize their performance and improve children health outcomes.

The study aimed to explore experiences of health care providers in disclosure of HIV positive status to the infected child and factors influencing the process.

## Methods

### Design

A qualitative design involving 20 healthcare providers from ten HIV care and treatment centers (CTC) was conducted from September, 2014 to October, 2014.

### Setting

The catchment area of the study was the Dar es Salaam Region of Tanzania. Tanzania is a country in East Africa. From the last census (2012), the country was estimated to have a population of about 45 million [15]. Dar es Salaam region has a population of about 4.3million people [15]. The study was conducted at ten Management and Development for Health (MDH) supported sites, which are HIV care and treatment clinics in Dar es Salaam, Tanzania. The MDH care and treatment program was established in 2004. The program provides infrastructure, laboratory, and technical support to the three municipalities (Temeke, Ilala and Kinondoni) in Dar es Salaam.

### Sampling and recruitment of study participants

Purposive sampling was used to select the ten CTC by considering the number of children living with HIV who attended the clinics. Healthcare providers experienced with paediatric HIV disclosure were selected deliberately so that they could contribute to the information needs of the study. The in-charge of these selected clinics were contacted by the researchers and were asked to identify health care providers who attend HIV infected children. Participants were included in the study if they met specific criteria : >18 years of age, had been practicing disclosure among HIV children for more than 6 months, and consented to participate in the study. Exclusion criteria included those <18 years of age, < 6 months experience in paediatric HIV disclosure and refusal to participate.

### Data collection procedures

A trained research assistant and one of the researchers conducted in-depth interviews using an interview guide translated into Kiswahili language. Eligible participants were given full explanation of the study and its importance. They were also informed of the data collection procedures which involved audio recording of in-depth interviews (IDIs). The interviews covered items in the interview guide (Table 1) plus additional information that was spontaneously suggested by participants. All participants responded to the questions asked during the interview.

**Table 1:** Sample interview questions

1. Tell me your experience with disclosure of child’s HIV positive status to the infected child. Probes:
a. How often do you do that? b. How old was the child/children? c. What made you disclose the child’s HIV positive status to the infected child? d. How do you conduct paediatric HIV disclosure? e. What challenges do you encounter during the process of paediatric HIV disclosure? f. What things or issues have ever made you fail to disclose to a HIV positive child about his/her status of being HIV infected? g. What did/do you fear most about paediatric HIV disclosure? h. How can these challenges be addressed?
2. Tell me your experience with paediatric HIV disclosure training the last time you attended it Probes:
a. How many days was it? b. What did you learn? c. Were you satisfied with knowledge and skills you got from the training? If not how would you like the paediatric HIV disclosure training to be conducted so you can be satisfied?

Field notes were taken throughout the interviews as a backup in case recording failed and to capture non-verbal information and key themes.

Interviews were conducted until no new information was obtained (data saturation) from participants and redundancy was achieved. The duration of each interview ranged from approximately 30 min to one hour.

### Ethical considerations

Ethical review and approval for human subject research was sought from Muhimbili University of Health and Allied Sciences (MUHAS). Participants who agreed to participate in the study were requested to complete the written informed consent form; only those who provided written consent were interviewed. Confidentiality and freedom to withdraw at any time during the study was ensured.

### Data analysis

Data was analyzed by a team of three researchers. An iterative approach of data analysis was used by looking at the data right from the time data was collected. Each day after data collection, the three researchers reviewed the recorded interviews and provided feedback to the interviewers. Audio-recorded interviews were transcribed, and translated into English. The researchers read the transcripts, discussed and agreed on the interpretations. Information from the field notes was expanded into rich descriptions and it was incorporated into the primary data. The interview transcripts were thoroughly evaluated to obtain an overall sense of the content of participants’ responses to various issues.

Data analysis followed content analysis (Graneheim & Lundman, 2004). Meaning units were identified from the transcripts and were condensed. Codes were developed from the condensed meaning units. They were sorted into categories which were later formulated into themes (Table 2).

**Table 2:** Example of analysis process

Meaning unit in the interview text	Condensed meaning unit	Code
There are challenges, there was one I wanted to disclose but the grandmother refused completely, she said her mother committed suicide the moment she knew her status, and when the father knew about the condition of the wife he ran away and until today he does not know how the child lives. I am also scared to tell the child because the child once told me that if my mother died, I will also die. So she is afraid to tell the child because the child might commit suicide as well, and the child has grown up	Fear of the child’s reaction after disclosure	Fear to disclose to the child, Fear of death, Uncertainty about HIV status disclosure, Uncertainty about child’s reaction
The training should teach about techniques to be used with children of different ages, also we should understand who is supposed to disclose to a child is it me or the parent? Because at other times we are told that a mother should start and then we should finish up, so maybe there is a mistake I do not know. At other times when you disclose directly to the child may be it is a mistake or maybe it is good, so I am kind of uncertain, is it I who should disclose or the mother	Lack of understanding about sequence of disclosing the HIV information to children and caregivers,	Confusion about disclosure process

### Trustworthiness

To ensure credibility of the study, healthcare providers with various experiences in paediatric HIV disclosure were purposively selected to participate in the study. In addition, data was collected using IDIs until no new information was obtained (saturation) from participants. Member checking was done throughout data collection to make sure that researchers correctly interpreted what participants reported. Selection of the most suitable codes and proper coverage of data by creating nodes, categories, and themes was also ensured.

To ensure dependability, researchers used the same interview guide to all participants to ensure consistency during data collection. Questions were re-phrased and modified as the researcher continued to collect data, to ensure that participants understood the information they were asked to give. Proper selection of participants, data collection and process of analysis plus appropriate quotations will allow readers to judge transferability of these findings.

### Results

Twenty (19 females and 1 male) healthcare providers from 10 care and treatment clinics were involved in in-depth interviews. There were 14 nurses, 4 nurse counselors and 2 medical doctors. All nurses had diploma in nursing. Nurse counselors had a degree in social work. One medical doctor had a degree in medicine while the other had an advanced diploma in clinical medicine. Participants’ ages ranged from 35 to 57 years, and working experience ranged from 1 to 6 years.

Health care providers’ experiences on paediatric HIV disclosure revealed four major themes (Table 3).

**Table 3:** Major themes of experiences on HIV paediatric disclosure among healthcare providers

Major Themes	
1.	Healthcare provider related factors
2.	Health system related factors
3.	Training and guideline related factors
4.	Parent/guardian related factors

In this study a guardian is defined as a person who looks after and is legally responsible for a HIV infected child whose parents have died or are not able to care for the child. Code numbers are used in the verbatim quotations to represent views from individual participants. A summary of quotes is provided on Table 4.

**Table 4:** Summary of quotes from the emerged themes

Thematic areas	Examples of quotes
Health care provider related factors	*“For the parent to be able to disclose, we first talk to the child. We then counsel the parents on how they can talk to the child”. N01*
	*“We need to understand who is supposed to disclose to the child; is it the healthcare provider or the parent/guardian?”Y01*
	*“Being healthcare providers, we are not supposed to disclose to the child. We should empower the parent/guardian so that she/he can disclose to the child.” L01*
	*“I often explain to the parent/guardian about the benefits of early disclosure. If she/he allows me to disclose to the child, I do it on the spot as long as the child has reached the required age.” T01*
Health system related factors	*“Each month we conduct a paediatric club in our clinic which enables us to identify children who are fully disclosed and those who are not.”L01*
	*We often use picture books to explain slowly to the child about the policemen (CD4 cells) in her/his body and their fight against the bad person (HIV virus). We tell the child, unless you take your medication, all the policemen in your body will be destroyed and you will become sick.” A01*
	*“We only have two picture books at the clinic. Sometimes I have to wait for my colleague to finish using the book before I proceed attending another child.” C02*
	*“When the child comes to the clinic, we ask if he/she is aware of her/his HIV status; if not we ask the parents/guardians to talk to the child.”Z02*
Training and guideline related factors	*“We are not skilled enough to disclose to children as we are for adults. Some of us have not received any training on paediatric HIV disclosure. This makes it difficult counselling parents/guardians about paediatric disclosure.” L01*
	*“In a one day paediatric HIV training we were encouraged to always ask what the child knows about HIV.” Y01*
	*Had we been adequately trained, we could have been be able to cope with challenges that we face when dealing with parents/guardians of these children. We need more skills on how to deal with these parents/guardians during the disclosure process. T01*
	*“It could be good if we received frequent training to update our knowledge on HIV issues concerning children.” C02*
Parent/guardian related factors inducted	*“Parents/guardians are often reluctant to disclose, they claim that the child is too young to be told about his/her HIV diagnosis. …Parents fear that the child might tell others in the streets…Others fear being hated by the child.” T01*
	*“Mothers find it difficult to disclose to their children because they feel guilty after having transmitted HIV infection to the child.” C01*
	*“Some parents find it easier lying about the diagnosis when confronted by their HIV infected children. A child may be told that he/she takes daily medication to treat conditions like TB or heart disease.” D02*

### Health care provider related factors

All healthcare providers reported to have engaged in the disclosure process. They declared that it was a good process, however they still face a lot of challenges. They claimed that children who are disclosed to early tend to understand better their disease condition and have improved drug adherence and health outcomes. In addition, healthcare providers reported that they initiate disclosure when a child persistently begins to ask questions, has poor drug adherence, and poor clinic attendance. Major challenges expressed by healthcare providers during the disclosure process included: negative emotional reactions from children, and parental refusal to disclose to the child.

One healthcare provider reported that:

*“Majority of parents/guardians are not ready to disclose when their children have reached the required age. Whenever they come to the clinic, we counsel them on how to disclose to their children but they do not want to do so.”T01*

The age of a child at which the process of disclosure was initiated varied among health care providers. Majority expressed that disclosure should begin gradually at an early age. Responses on the appropriate age to begin disclosure ranged from 3-8 years. Full disclosure was reported to occur between 9 and 14 years of age. In addition to a child’s age, they consider the child’s level of understanding about HIV and its treatment before fully embarking in the process. Some of the healthcare providers reported that during the initial stages of disclosure, they begin talking to the child before talking to the parent/guardian. They ask the child questions and establish rapport slowly until the child is ready for disclosure. A health care provider from one of the CTC clinics reported:

*“It is important to consider child’s age and level of understanding; we usually disclose to children aging 7 to 14 years.” G02*

Furthermore, the findings also show that healthcare providers are not sure about whose role it is to disclose to the child. Some expressed that, they are confused as to whether it is their responsibility or parents’ to disclose to the child the HIV-positive status.

### Health system related factors

Some of the healthcare providers reported using books with pictures, models, and drawings to aid in the process of disclosure. For example, one of the books used is about a policeman. The healthcare providers talk to the child about the policeman in the book and his role in protecting citizens. They then link the story with the child’s condition explaining the role of medication in protecting the child’s body. They keep telling the same story to the child until they feel that the child is ready to be disclosed to. Health care providers from clinics that did not have books reported drawing pictures or using other examples during the process of disclosure.

Healthcare providers from different sites reported using different mechanisms of follow-up. They conducted clubs, and used stickers or letters on the files of those disclosed and non-disclosed. This enabled them to follow up HIV infected children for further disclosure, management and care:

One healthcare provider reported:

*“We usually mark with a letter “D” on top files of children who have been fully disclosedThe rest with no Ds are reminders that we need to follow up the children making sure that they are disclosed to.”L01*

### Training and guideline related factors

Findings show that, healthcare providers differed in the way they disclosed the HIV-positive status to the infected child. Others were aware of the national guidelines for paediatric HIV disclosure which requires the parent/guardian to disclose to the child. However, some reported to have conducted the disclosure themselves because of parental refusal to do so. Others were completely unaware of the guidelines and they disclosed to the child directly. A health care provider expressed that:

*“I make sure I disclose to all the children if they have reached the required age.”T01*

Another one from a different clinic reported that:

*“We have been taught from the national guidelines for paediatric disclosure that parents should start disclosing to the child gradually.”G02*

Lack of paediatric HIV disclosure training and different sources of training for some of the healthcare providers were seen as reason for this uncertainty. Findings revealed that healthcare providers have received training about disclosure from a variety of sources; however, these trainings were not standardized. In addition, the training content stipulates different ages to begin the disclosure process; ranging from 3–8 years of age. One health care provider expressed:

*“I have acquired the ability to disclose from different HIV trainings Last time I attended a two weeks pediatric HIV training and I learned that disclosure can begin when the child is six years of age.”T01*

Other healthcare providers expressed that they are more skilled in adult HIV disclosure than paediatric HIV disclosure, making it more difficult to support parents/guardian in disclosing to the HIV infected children. This fact adds more difficulties in their ability

Healthcare providers who had been trained declared that their responsibility was to support parents so that they can disclose HIV-positive status to the infected child. However, others complained that they were confused and did not know if it was them or the parent/guardian who should make the disclosure to the child. This shows a discrepancy in the types of training that healthcare providers have received regarding disclosure of HIV positive status to the infected child.

They thus suggested that further training on how to conduct paediatric HIV disclosure is required.

In addition, health care providers suggested that the duration of training should be longer and the training curriculum should include child psychology topics and different techniques on how to support parents/guardians in the disclosure process.

*The duration of training is too short and only a few of us have been trained. Other healthcare providers learn from those who have already been trained. D01. D01*

The healthcare providers recommended that paediatric HIV disclosure training should focus more on practical skills rather than theory so that they can acquire the ability to conduct disclosure more effectively. In addition, they suggested that more efforts should be put on empowering parents/guardians in paediatric disclosure techniques with emphasis on early disclosure.

*We need to be trained for at least two weeks; one week for theory and another one for practical skills. K01*

### Parent/guardian related factors

In order to improve the ability of parents/guardians to disclose HIV-positive status to the HIV infected child, some of the healthcare providers reported providing education and counseling sessions to parents/guardians on the importance of early disclosure. In addition, they provided assistance to parents/guardians during the disclosure process. They emphasized the benefits of parents/guardians disclosing to children early to foster adherence to treatment, support the child, help the child cope, increase child’s understanding about their disease, and protect others from HIV infection. Parents/guardians were empowered to begin disclosing to the child slowly at a younger age to help prepare the child psychologically for full disclosure.

*Parents should start talking to their child when he/she is 6 years old. We often counsel parents so that they can begin to disclose to their children gradually. A child should be aware of his/her problem when he reaches 8 years of age. Z02*

Healthcare providers reported facing a lot of problems when they supported parents in the disclosure process. Most parents/guardians are not ready to disclose to the child because of fear of stigma. Parents have always been hesitant to disclose to children because of fear that the child will spread news in the streets about their HIV infection. They also fear that the child might not react well and perhaps not do well in the studies. Other parents do not want their child to be told because they believe they are too young to understand and handle such information:

*Example there was one parent who wanted us to wait until her child completed examinations because she thought the child would be confused.” C02*

Another healthcare provider from a different clinic commented:

*“Majority of parents fear telling the child because they believe he/she will not be happy. We therefore counsel the mother on how she can disclose to her child.” K01*

In addition, parents reported more guilt in comparison to guardians when it came to disclosure. Parents felt that they were the source of infection which made it more difficult for them to share the diagnosis and receive support from healthcare providers.to support them on early disclosure. Healthcare providers reported that compared to guardians, most parents do not want to disclose the HIV status to the infected child. They prefer the process to be carried out by healthcare providers to save them the trouble of being blamed and hated. Delay in paediatric HIV disclosure made it more difficult for parents to disclose to older children because of the anticipated negative emotional reaction once disclosed to.

*It is very difficult for a parent telling her own standard one child that: “You are HIV-positive and I am the cause for the problem.” C01*

Healthcare providers expressed their concern about the fact that parents/guardians hide the truth about child’s HIV positive status. Instead, parents/guardians were reported to tell the child that he/she has other medical conditions to justify daily use of medication.

## Discussion

The study provided a broad view on health care providers’ experiences with the disclosure process in children infected with HIV. Various factors were shown to influence paediatric disclosure including health care provider related factors, parent/guardian related factors, health system related factors, and training and guideline related factors.

### Healthcare provider related factors

Findings show that, healthcare providers have different views and experiences regarding who is right person to tell the child about his/her infection status. Some of the healthcare providers carried out the process themselves while others, perceived that it was the parents/guardian who should disclose to the child. Studies have shown variations of perspectives regarding disclosure to HIV infected children [16, 17]. It was also revealed by healthcare providers that timing for disclosure was a challenge. Studies have reported different results regarding the right time at which paediatric HIV disclosure should begin [18].

Some of the healthcare providers pointed out that their role was to support parents/guardians in the disclosure process but not to disclose to the child. They believed that the caregivers should lead the process because they are in a better position to support the child in adhering to the prescribed treatment regimen and provide support and comfort to the child after disclosure [16]. However, guardians/parents reported delaying paediatric HIV disclosure because they felt they had inadequate knowledge and skills to disclose. Most parents/guardians sought the support of healthcare providers during the disclosure process [19]. Similar to other studies, healthcare providers in this study were reported supporting parents/guardians by providing education and counseling during and after paediatric HIV disclosure [16]. Healthcare providers reported that they educated and counseled parents/guardians on early disclosure during the club sessions that were conducted once each month. A similar study in Kenya showed that during clinic days, healthcare providers counseled parents/guardians in the process of disclosure which played an important role in preparation and readiness for the disclosure to their children.

### Health system related factors

A few healthcare providers reported using models and pictures during the disclosure process. These supporting tools were only found among the few healthcare providers who had undergone paediatric HIV training. Lack of supportive educational materials at the health facility has been found to increase healthcare provider constraints in the process of disclosure [18].

### Training and guideline related factors

The national paediatric HIV disclosure guidelines stipulate that healthcare providers should counsel parents/guardians on how to disclose to the HIV infected children. In order to do this healthcare providers need to be equipped with knowledge and skills on Paediatric HIV disclosure counseling. However, findings from this study shows that most healthcare providers are not certain as to who should tell the child that she/he is infected with HIV and they do not have a specific time to initiate the disclosure process. This discrepancy may have been contributed by complete lack of standardized national training for paediatric HIV disclosure that varied in content and duration [16, 20]. Similar studies have reported missing contents in paediatric HIV disclosure training including on the appropriate age and maturity level to initiate the process of disclosure [21]. WHO has recommended that paediatric HIV disclosure training for healthcare providers should cover the following vital areas: stigma, privacy, culturally appropriate disclosure process, communication based on child’s age, HIV information to parents/guardians and children, emotional preparation and management, responding to children’ concerns, and planning for the future [22].

Healthcare providers expressed the need to be trained on paediatric HIV disclosure because the majority had not undertaken such training apart from on job training. This is consistent with other studies that have reported the need for ongoing training, counseling and debriefing to deal with HIV infected children [16]. Similar views from another study of healthcare providers in Kenya and Uganda revealed that majority of them have had limited training in paediatric HIV disclosure [18, 23], which leads to a lack of confidence and inability to handle these situations. Improved training on paediatric HIV disclosure would alleviate the discrepancies that exist among healthcare providers regarding whose responsibility it is to disclose to the HIV infected child.

In addition, healthcare providers suggested that there should be appropriate guidelines on paediatric HIV disclosure particularly on the age when disclosure should begin. Consistent with other studies, the majority of healthcare providers also suggested that they need to be equipped with educational materials such as posters and other reference guidelines on paediatric HIV disclosure [18].

### Parent/guardian related factors

Studies have shown that HIV infected children whose status is disclosed to them by their parents/guardians tend to have improved decision making abilities, can protect themselves and others from further HIV infection, show cooperation in treatment, and have a better understanding about their illness [24]. The role of healthcare providers in paediatric disclosure has been described in other studies. In some studies other healthcare providers perceive that it is their role to support parents/guardians in leading the disclosure process while others lead and initiate paediatric HIV disclosure themselves [16].

Health care providers reported that most parents/guardians have not been exposed to disclosure counseling which makes them hesitant to disclose to their children. Thus some healthcare providers focused on educating and supporting parents/guardians in order to empower them to talk to their children about the HIV positive status [24].

Findings show that healthcare providers face a lot of challenges as they support parents/guardians in the disclosure process. This is consistent with other studies that have documented difficulties in disclosure because parents/guardians think that the child is too young to understand what it means to have HIV [25] and believe that it is not the right time to tell the child [19]. Other barriers to disclosure that were reported by healthcare providers include: parents’ fear of being blamed by their children; parents feeling guilty due to the societal perception of HIV transmission and fear that the child will tell others about the HIV diagnoses [24, 26]. The parent/guardian believe when the child is too young he/she is not capable of keeping secrets, hence the parent/guardian often chose to wait until the child is older [19]. Once the child tells others, the parents fear they will be discriminated against, rejected, and laughed at because of the social stigma surrounding the HIV diagnosis [26].

In addition, parents/guardians fear that the child could have negative emotional consequences after their diagnosis has been disclosed to them. Similar studies [27, 16, 21, 19, 28], have reported that parents fear that the child will cry, be sad, run away, harm themselves and lose hope.

Healthcare providers reported that parents/guardians found it difficult to disclose to their children because they did not know how to tell. This could have been contributed to by the lack of skills and knowledge necessary to handle the disclosure process and dealing with the negative consequences. Healthcare providers reported that parents often asked them to disclose to the child instead. Consistent with other studies, some parents/guardians resorted to deception as a way of coping with questions from their children. This delays disclosure until the parent/guardian deems they are ready [20]. An example of deception was telling the child that he or she has a chronic illness to justify why the child takes daily medication.

### Limitations

We were able to obtain perspectives of healthcare providers regarding paediatric HIV disclosure which depicted confusion about their role during the process. However, we could not explore more on the perspectives of parents/guardians regarding their role in paediatric HIV disclosure.

## Conclusion

This study has highlighted the process of disclosure in children living with HIV from a healthcare provider perspective. The study has highlighted the complexity of paediatric HIV disclosure process among healthcare providers. Confusion about their role and responsibility of healthcare providers during the process of disclosure was a major constraint reported. This was largely related to inadequate training, and lack of clear guidelines and unstandardized training which differ in duration and content. Parents/guardians were reported to lack knowledge and skills in disclosing the HIV status to the infected child. Provision of standardized paediatric HIV disclosure training with more emphasis on practical skills, increased training duration, and inclusion of disclosure content that is age appropriate for children with HIV would help prevent inconsistencies during the disclosure process. In addition, reviewing the current standard operating procedures for paediatric HIV disclosure would improve support for parents/guardians in disclosure of HIV-positive status to the infected child. This in turn would facilitate child’s adherence to the treatment regimen.

## Abbreviations

AIDS: Acquire immuno-deficient syndrome
CTC: Care and Treatment Centre
HIV: Human immuno-deficient virus
IDIs: In-depth interviews
MDH: Management and development for health
UNAIDS: United Nations acquire immuno-deficient syndrome
